# Audit of Pre-Treatment Baseline Investigations for Patients Started on Atypical Antipsychotics – Department of Psychiatry, Gulab Devi Teaching Hospital, Lahore

**DOI:** 10.1192/bjo.2026.11839

**Published:** 2026-06-30

**Authors:** Muhammad Mansoor Ali, Syeda Areeba Siraj, Mudassar Ijaz, Ali Abbas Zahid, Maryam Arshad

**Affiliations:** 1 Al Aleem Medical Collge / Gulab Devi Teaching Hospital, Lahore, Pakistan; 2 Avicenna Hospital, Lahore, Pakistan

## Abstract

**Aims:**

To assess compliance with recommended baseline physical, laboratory, and additional investigations prior to initiation of atypical antipsychotics in psychiatric inpatients, identify deficiencies in practice, implement targeted interventions, and evaluate subsequent improvement in adherence.

ET AL AUTHORS : Dr.Subhash Chandar (Email: dr.sbhalwani@gmail.com) - Psychiatry PGR Suleman Roshan Medical College,Tando Adam, Sindh, Pakistan.Dr Muhammad Zubair (Email: Muhammadzubair1103@gmail.com) - General Practitioner

**Methods:**

A prospective closed-loop clinical audit was conducted in the Psychiatry Department over two months, comprising pre- and post-intervention phases. A total of 50 patients newly started on atypical antipsychotics were included. Baseline parameters assessed were:

Physical: weight/BMI, waist circumference, blood pressure

Laboratory: complete blood count (CBC), liver function tests (LFTs), renal function tests (RFTs), fasting blood glucose or Hbs-738c, fasting lipid profile, creatine phosphokinase (CPK), serum prolactin

Additional: ECG and pregnancy test (females of reproductive age)

Pre-intervention data were collected via review of medical records. Interventions included staff education, introduction of standardized baseline investigation checklists, visual reminders, and improved coordination with laboratory and ECG services. Post-intervention data were collected using the same proforma to assess improvement.

**Results:**

Pre-intervention (n=25; 10 males, 15 females):

Weight/BMI was documented in 16 patients (64%), blood pressure in 7 (28%), and waist circumference in none (0%); no physical assessments were recorded in 7 patients (28%). Laboratory investigations showed low compliance: CBC in 14 (56%), LFTs and RFTs in 13 each (52%), glucose/HbA1c in 11 (44%), lipid profile in 12 (48%), while CPK and prolactin were not documented in any patient; no laboratory investigations were recorded in 11 patients (44%). ECG was performed in 10 patients (40%), and no pregnancy testing was documented.

Post-intervention (n=25; 9 males, 16 females):

Weight/BMI documentation increased to 17 patients (68%), blood pressure to 19 (76%), and waist circumference to 12 (48%), with no physical assessments missing in 80% of patients. Laboratory monitoring improved markedly: CBC 25 (100%), LFTs 22 (88%), RFTs 21 (84%), glucose/HbA1c 19 (76%), lipid profile 15 (60%), CPK 12 (48%), and prolactin 15 (60%), with only 2 patients (8%) lacking laboratory investigations. ECG documentation increased to 17 patients (68%) and pregnancy testing to 14 females (88%).

Overall, physical assessments improved from 30.7% to 64%, laboratory investigations from 36% to 76.6%, and additional investigations from 20% to 62%. Combined compliance improved from approximately 29% pre-intervention to 68% post-intervention, representing an absolute improvement of 39 percentage points.

**Conclusion:**

Baseline monitoring prior to initiation of atypical antipsychotics was initially suboptimal. Implementation of simple, low-cost interventions resulted in substantial improvements across physical, laboratory, and additional monitoring domains, demonstrating the effectiveness of structured quality improvement measures in enhancing patient safety and adherence to recommended standards.

